# Insights into medication-induced liver injury: Understanding and management strategies

**DOI:** 10.1016/j.toxrep.2025.101976

**Published:** 2025-03-01

**Authors:** Vatsalya Tiwari, Shrishti Shandily, Jessielina Albert, Vaibhav Mishra, Manoj Dikkatwar, Rohit Singh, Sujit Kumar Sah, Sharad Chand

**Affiliations:** a Amity Institute of Pharmacy, Amity University Uttar Pradesh, Noida, India; b DY Patil University School of Pharmacy, DY Patil (Deemed to be University), Nerul, Navi Mumbai, Maharashtra 400706, India; c Department of Pharmaceutical Sciences, School of Health Sciences and Technology, Dr. Vishwanath Karad MIT World Peace University, Pune, Maharashtra 411038, India

**Keywords:** Acute liver toxicity, Cytochrome P-450 enzyme, Drug metabolism, Hepatotoxicity, Liver failure

## Abstract

Drug-induced liver injury (DILI) has increasingly become a major concern in Western countries since the late 1960s, with an estimated annual incidence of 13.9–19.1 cases per 100,000 people. DILI is a significant cause of acute liver failure, exhibiting a high mortality rate of 10–50 %. Its etiology includes medications, herbal products, and dietary supplements, exacerbated by pre-existing liver conditions, sonorities, pregnancy, and nutritional deficiencies. It is categorized into intrinsic and idiosyncratic reactions. Intrinsic DILI, dose-dependent and predictable, is primarily caused by substances like paracetamol, which leads to liver toxicity through direct metabolic pathways. In contrast, idiosyncratic DILI is less common, unpredictable, and affects susceptible individuals, with non-steroidal anti-inflammatory drugs, antibiotics, and cardiovascular agents frequently implicated in hospitals. Oxidative stress, mitochondrial dysfunction, bile salt export inhibition, and stress on the endoplasmic reticulum are some DILI-related pathophysiology. Diagnosis relies on biochemical tests, serological markers, radiological investigations, and liver biopsy. Management strategies emphasize the identification and cessation of the offending drugs, supportive care, and specific treatment options targeted to the culprit drugs. Management depends on the severity and nature of the injury.

## Highlights

• The spectrum of DILI encompasses around 1/5th of total population prescribed with medications.
• It causes acute liver failure with significant morbidity and mortality.
• Intrinsic DILI is dose dependent and idiosyncratic is usually unpredictable.
• Diagnosis incudes laboratory test; management includes cessation of culprit drugs and supportive cure.

## Graphical Abstract

**Figure:**
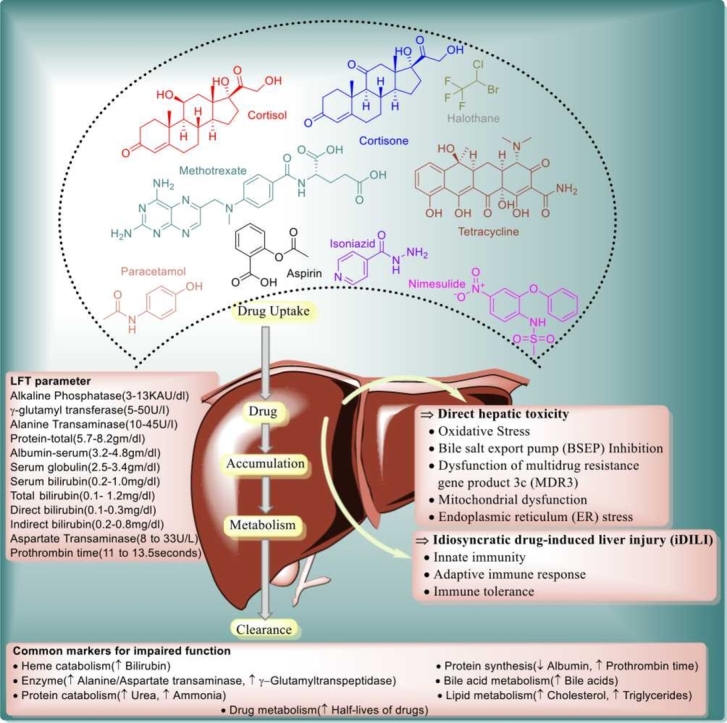
Medication-induced liver injury: Drugs, mechanism and clinical presentations.

## 1 Introduction

Drug-induced liver injury (DILI) cases have increased since the late 1960’s. The estimated annual incidence ranges between 13.9 and 19.1 cases per 100,000 people [1]. In Western countries, DILI is the most prevalent reason for acute live failure (ALF) with a mortality rate of 10–50 % of cases [2]. The frequent causes of DILI are medications, herbal products, and dietary supplements. Further, the occurrence of DILI is increased among individuals with presented liver diseases, comorbidities, pregnancy, and poor nutritional status which impair the ability to metabolize medications adequately [2], [3]. DILI is either an intrinsic or idiosyncratic reaction. Intrinsic DILI is majorly dose-dependent directly causing liver toxicity. Paracetamol overdose is commonly reported as causing intrinsic DILI while tetracycline, aspirin, and vitamin A are rarely causing it [3]. In idiosyncratic DILI, non-steroidal anti-inflammatory drugs (NSAIDs), antibiotics, cardiovascular agents, herbal products, and dietary supplements are documented as more frequently causing agents. Upon its occurrence, in the majority of cases the administrated culprit drugs need to stop and normalize the elevated liver enzymes with supportive treatment, after cessation of toxic drug treatment [4].

## 2 Types of DILI

### 2.1 Intrinsic DILI

Intrinsic DILI is dependent on doses and can be predicted when an individual ingests a sufficiently high quantity of drugs that have the potential to deposit in the liver and induce various degrees of liver injury within hours or days of exposure. It is believed that the drug metabolites undergo the process of biomodification in the liver and are converted into reactive metabolites resulting in oxidative stress and hepatic cell damage. Acetaminophen is the most used medication that causes intrinsic DILI while other drugs causing Intrinsic DILI are presented in Table 1 [4], [5], [6].

**Table 1:** Drugs Associated with Liver Injury [1], [2], [3], [4], [5], [6].

### 2.2 Idiosyncratic DILI

**Fig. 1:**
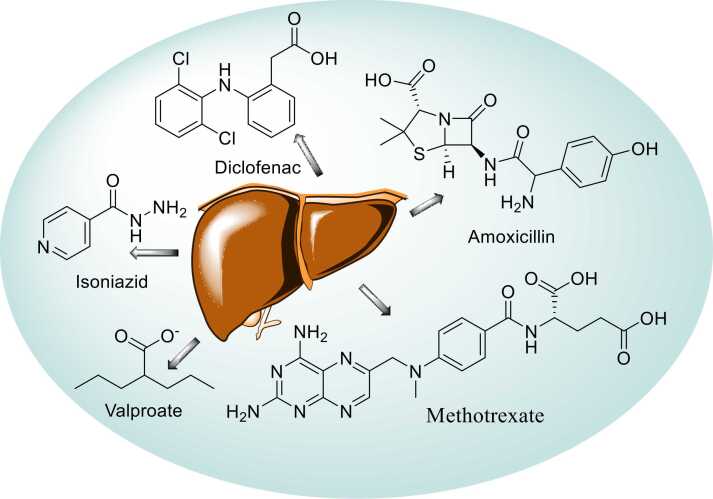
Drugs responsible for Idiosyncratic DILI.

DILI is less common, unpredictable, observed within weeks to months of exposure and affects only susceptible patients. Idiosyncratic DILI varies in presentation, course and immune activity and is not consistently related to medication dose. The common drugs that induce idiosyncratic DILI are amoxicillin-clavulanate, diclofenac, statins, isoniazid, Valproate and methotrexate [5], [6]. Fig. 1

## 3 Pathophysiology of drug metabolism

### 3.1 Normal drug metabolism and transport

The liver metabolizes the ingested medications. Drug molecules are passively taken up by hepatocytes or transported into hepatocytes through various transport proteins in the sinusoidal plasma membrane. Large protein families in the liver regulate drug absorption, drug distribution, drug metabolism and drug disposal. Metabolism of disposed drug initiated by Phase I reaction and Phase II reaction [7], [8], [9], [10].

### 3.2 Drug metabolism pathways

#### 3.2.1 Phase I pathway

The family of cytochrome 450 enzymes mediated the drug-metabolizing enzymes in phase I reaction including oxidation, reduction, and hydrolysis, and converted to drug metabolites. Further, the CYP450 enzyme transformed drug metabolites into water-soluble byproducts [11], [12].

Cytochrome P450 iron porphyrin proteins (CYP) are significant players in oxidative and reduction reactions required for drug metabolism.

#### 3.2.2 Phase II pathways

In the phase II reaction, the presence of transferase enzymes, drug metabolites byproduct from Phase I are enzymatically conjugated with glucuronidation, acetylation, and sulfation which are hydrophilic endogenous molecules that enhance the water solubility and facilitate the excretion of drug metabolites through Bile [11], [12].

#### 3.2.3 Phase III pathways

Large and/or ionized molecules require carrier-mediated transport proteins for movement into and out of cells. These transport proteins belong to two major families: solute carriers (SLC) and ATP-binding cassette (ABC) transporters. The SLC family includes transporters such as organic anion transporters (OATs), organic cation transporters (OCTs), and organic anion-transporting polypeptides (OATPs), which primarily mediate the uptake of small molecules into cells [13], [14].

In contrast, ABC transporters play a crucial role in the efflux of large or ionized molecules out of cells. Together, these transporters constitute the Phase III pathway, which is integral to the elimination and transport of xenobiotics and endogenous compounds [12], [13], [14], [15], [16].

### 3.3 Pathophysiology of DILI

The liver plays a crucial part in biotransformation and eliminating medications from the bloodstream. According to the drug's hepatotoxicity, DILI is typically categorized into two subtypes: idiosyncratic and intrinsic hepatotoxicity. The dose-dependent hepatotoxicity can be predicted in people or animal models, whereas idiosyncratic DILI is unpredictable and cannot be explained by known pharmacological features [17], [18]. DILI typically results from the pathophysiology of a harmful substance or metabolite that either triggers an immediate immune reaction or alters the cell's biochemistry. In either scenario, the subsequent cell death is what causes the clinical hepatitis presentation [19].

#### 3.3.1 Direct hepatic toxicity

Cytochrome P450 iron porphyrin proteins (CYP) are significant players in oxidative and reduction reactions required for drug metabolism, leading to the formation of polarized ions, oxygen free radicals, and more reactive compounds. These reactive metabolites can alter the function of cellular proteins by covalently binding to them or by producing immunogenic haptens, which provoke an immunological response [6], [16]. Phase I–III reactions are collectively known as detoxification processes, primarily involved in the inactivation of toxic compounds or toxicants. However, when the quantity of these compounds exceeds the hepatic detoxification capacity, it disrupts hepatic cell function, triggering apoptotic or necrotic cell death, which can range from mild to severe tissue damage. Paracetamol is the most extensively studied drug for its ability to induce intrinsic DILI [16], [20]. Moreover, alterations in mitochondrial permeability transition (MPT) can lead to disruptions in cellular energy homeostasis. MPT plays a critical role in regulating cell death pathways, including both apoptosis and necrosis. In apoptotic cell death, MPT is associated with the release of cytochrome c and the activation of downstream caspases. However, under conditions of severe oxidative or toxic stress, sustained MPT can lead to necrotic cell death due to ATP depletion and membrane rupture [6], [16], [20].

##### 3.3.1.1 Oxidative stress

Production of reactive oxygen species (ROS) build-up by the redox process in the liver during drug metabolism contributes to DILI through several mechanisms [21]. The free radical metabolites can progressively damage cells by covalently binding to macromolecules, leading to their accumulation in the liver. Additionally, these reactive components can oxidize critical cellular structures, resulting in alterations to mitochondrial functions and genomic DNA. Specifically, oxidative damage may influence the expression and activity of key regulatory proteins like p21 and p53, which are involved in cell cycle regulation and apoptosis. This disruption may promote cell necrosis and, in some cases, contribute to tumorigenesis [22]. Furthermore, oxidative stress and mitochondrial dysfunction may also trigger epigenetic modifications, such as altered DNA methylation or histone acetylation, which further exacerbate cellular damage. Lastly, disruption of the mitochondrial membrane permeability transition (MPT) contributes to mitochondrial swelling, loss of membrane potential, and eventual cell death [18].

##### 3.3.1.2 Bile salt export pump (BSEP) Inhibition

BSEP is encoded by the ABCB11 gene expressed on the canalicular membrane of hepatic cells. The inhibition of BSEP by drug resulted in high bile salt concentration inside hepatic cells that can harm mitochondria, and transporter tubules and cause cytotoxicity leading to DILI. Bosentan and cyclosporine drugs are examples of BSEP inhibitors. According to the literature, drugs that potentially block the BSEP may increase the risk of developing idiosyncratic DILI than the drugs that do not block BSEP [23], [24].

##### 3.3.1.3 Dysfunction of multidrug resistance gene product 3c (MDR3)

MDR3 encoded by the ABCB4 gene, moves the phosphatidylcholine chemical [25], a substance found in eggs, soybeans, mustard, sunflower and other foods on the inner to the outside leaflet of lipid bilayer which results in various types of cholestasis [20]. If bile secretion is blocked, basolateral MRP3 expression in hepatocytes permits the efflux of organic anions from the liver into circulation, indicating that MRP3 serves as a backup route for amphipathic anions under cholestatic conditions [26]. During cholestasis, basolateral MRP3 expression is increased [27].

##### 3.3.1.4 Mitochondrial dysfunction

Hepatocellular necrosis is mostly caused by dysfunctional mitochondria. The production of mitochondrial components is inhibited, and mitochondrial membrane permeability transition (MPT) is accelerated due to increased oxidative stress. This enables the departure of smaller molecules of less than 1500 Daltons from mitochondria which further depolarizes the mitochondria and lowers the proton gradient leading the mitochondrial membrane to collapse. The next step is for the mitochondria to enlarge, burst and release proteins into the intermembrane gap. This action is related to cell death mechanisms like apoptosis [18], [28], [29].

##### 3.3.1.5 Endoplasmic reticulum (ER) stress

ER stress is one of the major factors in drug-induced hepatotoxicity and can be triggered by several cellular stressors, including ROS or changes in the calcium (Ca^2 +^) concentration within cells. The formation of reactive metabolite species from the metabolism of the drugs causes ROS overproduction, mitochondrial malfunction, MMP loss and increased intracellular Ca^2+^ concentration could all contribute to ER stress [16], [20].

#### 3.3.2 Idiosyncratic drug-induced liver injury (iDILI)

iDILI (idiosyncratic drug-induced liver injury) is an atypical pharmacological adverse reaction that may manifest weeks or months after the ingestion of an offending drug, though it can also occur unexpectedly during drug therapy [5], [12], [20]. Its occurrence is closely linked to the host’s health status, behavioural factors, and exposure to certain substances. Behavioural factors, such as smoking and excessive alcohol consumption, are known to increase the risk of iDILI by amplifying oxidative stress and impairing normal liver function. Both genetic and non-genetic factors contribute to iDILI susceptibility. Genetically, iDILI is associated with abnormal immune responses, such as a loss of immune tolerance, and reduced activity of drug-metabolizing enzymes. Non-genetic factors include underlying health conditions, pregnancy, age, and gender. These factors may affect liver resilience and the ability to manage drug-related stress. The pathophysiology of iDILI involves mechanisms similar to intrinsic DILI, including the overproduction of reactive oxygen species (ROS), mitochondrial dysfunction, and disruption of bile acid homeostasis. In certain cases, iDILI is also linked to the liver’s production of neoantigens in response to specific drug exposures. These neoantigens can trigger an idiosyncratic immune response by activating immune cells, leading to liver injury. This immune response reflects a breakdown in immunotolerance, where the immune system mistakenly recognizes the drug or drug-protein adducts as harmful, resulting in an inflammatory attack on the liver [4], [5], [18].

##### 3.3.2.1 Innate immunity

The innate immune system in the human liver is mostly composed of Kupffer cells (KCs), neutrophils, monocytes and natural killer cells/natural killer T cells (NK/NKT cells) [18]. Antigens from damage-associated molecular patterns (DAMPs) bind to the Toll-like receptors (TLRs), scavenger receptors (SCRs) and mannitol receptors (MRs) on macrophages, activating innate immune system cells and inducing inflammation [18], [30].

##### 3.3.2.2 Adaptive immune response

The liver damage returns after a known iDILI patient is exposed to the culprit drug as soon as possible again suggesting that an adaptive immune response is involved. Drug metabolites form protein haptens that covalently bind to hepatic proteins or modified proteins expressed cell membrane of hepatocytes during the drug metabolism process. Sulfamethoxazole, lamotrigine and carbamazepine are examples of common drugs known to cause adaptive immune response [31].

##### 3.3.2.3 Immune tolerance

In majority of patients exposed to the culprit drug, especially at high doses stress in hepatocytes increased. However, only a very small percentage of people experience injury. Despite the liver's thought to possess immunological tolerance, there are still some people who are more susceptible to hepatocyte stress resulting in DILI occurrence. The apoptosis process due to activated T cells, immunological deviation and immune active suppression can be a reason for the immunity-related tolerance phenomenon [5], [18].

## 4 Some of the common drugs causing the DILI

### 4.1 Paracetamol

Paracetamol is the most widely used drug as an antipyretic and analgesic. In liver microsomes, 5–10 % of a therapeutic dose of paracetamol is converted into the reactive metabolite **N-acetyl-para-benzoquinone imine (NAPQI)** by cytochrome P450 isoforms (primarily **CYP2E1, CYP1A2, and CYP3A4**), which are predominantly associated with paracetamol-induced hepatotoxicity [3]. The hepatotoxicity caused by NAPQI is directly linked to the excessive accumulation of paracetamol in hepatocytes. At toxic doses, most of the drug is metabolized via the CYP2E1 pathway, resulting in the formation of toxic concentrations of NAPQI. This leads to **glutathione (GSH) depletion**, activation of GST-S-transferases, cessation of ATP generation, formation of protein adducts, and altered mitochondrial function [32]. These mitochondrial changes disrupt cellular homeostasis, increasing cell membrane permeability, causing cellular swelling, karyolysis, vacuolization, and loss of organelles, ultimately leading to hepatocyte necrosis [33].

Under normal (non-toxic) conditions, hepatic glutathione detoxifies NAPQI by forming conjugates through **glutathione-S-transferase (GST)-mediated reactions**. Additionally, paracetamol is metabolized predominantly via **glucuronidation and sulfonation reactions** in Phase II, producing non-toxic metabolites. During the biotransformation process, NAPQI conjugates are converted into **mercapturic acid and cysteine complexes**, which are excreted through urine [34].

### 4.2 Nonsteroidal anti-inflammatory drugs except paracetamol and aspirin

NSAIDs are recommended or used in the pharmacotherapy of several headaches, fever and acute and chronic inflammatory disorders. Idiosyncrasy rather than intrinsic toxicity, appears to be the mechanism by which practically all NSAIDs cause liver damage. Acetaminophen and aspirin are the main exceptions to this rule, which result in a dose-dependent hepatic injury. Although there is evidence of an immunologic cause in many NSAID-related liver injury, there is evidence that some NSAIDs' toxic metabolites contribute to liver injury [35].

### 4.3 Aspirin

Although salicylate poisoning causes a variety of metabolic abnormalities, the main pathophysiologic mechanism is an alteration in aerobic metabolism caused by the uncoupling of mitochondrial oxidative phosphorylation. Several mitochondrial processes that are mediated by enzymes are disrupted as a result [36]. A distinctive form of hepatotoxicity is Reye's syndrome, lactic acidosis development, aspirin use of micro vesicular fat and liver malfunction accompanied by encephalopathy comatose [37]. Serum aminotransferase levels are typically abnormally elevated, but low serum bilirubin levels or worsened liver failure symptoms, such as hyperammonemia and encephalopathy. It appears in young adults or children who suffer from febrile sickness, usually a few days to a week following chickenpox or influenza B. Although mild cases tend to recover rapidly, severe cases may be often fatal. Serum aminotransferase levels are usually high despite liver failure including hyperammonemia and encephalopathy, serum bilirubin levels are either low or slightly elevated [37], [38]. The link of hepatotoxicity with high-dose aspirin uses and short latency periods formed direct endogenous hepatotoxin. Individuals with severe liver due to aspirin can use acetaminophen, other NSAIDs, and low doses of aspirin without problems [39].

### 4.4 Nimesulide

It is repeatedly linked with clinically evident liver injury along with jaundice. The period of onset ranged from a few days to 6 months. The enzyme elevations are typically hepatocellular, although cholestatic forms have also been reported. Immune-allergic features are usually absent or, if present, unremarkable. The mechanism of nimesulide-induced hepatotoxicity is unknown, but researchers believe an idiosyncratic reaction due to its intermediate metabolism [40].

### 4.5 Methotrexate

Methotrexate is an antimetabolite and folic acid antagonist that is widely used to treat rheumatoid arthritis, leukemia, lymphoma and many solid organ cancers. Prolonged use of methotrexate therapy has demonstrated the ability to reduce serum aminotransferase enzyme and is associated with the development of fatty liver disease, fibrosis and cirrhosis. Methotrexate in hepatocytes is thought to cause harm directly through the suppression of RNA and DNA synthesis in the liver resulting in cellular arrest. Methotrexate therapy leads to the elevation of hepatic stellate cell numbers which initiate liver fibrosis but the exact mechanism of methotrexate causing liver fibrosis is unclear [41], [42].

### 4.6 Corticosteroids

Prolonged corticosteroid use is associated with a variety of hepatic effects, including **hepatomegaly**, **glycogenosis**, and **steatosis**. In individuals with chronic liver conditions, such as viral hepatitis, long-term corticosteroid therapy may worsen the preexisting liver damage. Abrupt discontinuation of corticosteroid pulse therapy may result in **reactivation of Hepatitis B** or **exacerbation of autoimmune hepatitis**, both of which can be life-threatening. However, these effects are distinct from the mechanisms underlying corticosteroid-induced **drug-induced liver injury (DILI)** [43].

**Mechanisms of Corticosteroid-Induced DILI:**

a) **Mitochondrial Dysfunction**: Corticosteroids can impair mitochondrial function in hepatocytes by disrupting the electron transport chain, leading to **reduced ATP production**. This dysfunction contributes to the generation of **reactive oxygen species (ROS)**, which causes oxidative stress and subsequent hepatocyte damage.
b) **Oxidative Stress**: The accumulation of ROS leads to lipid peroxidation of cellular membranes, protein oxidation, and DNA damage in hepatocytes. This oxidative stress can activate signalling pathways that result in hepatocyte apoptosis or necrosis, depending on the severity of the insult.
c) **Immune Modulation and Inflammatory Response**: Corticosteroids, while often used to suppress inflammation, can paradoxically disrupt immune homeostasis in certain cases. This dysregulation may exacerbate an underlying inflammatory process, leading to enhanced hepatocyte damage. In some patients, corticosteroids may also modulate the expression of **pro-inflammatory cytokines** or impair the clearance of toxic substances, compounding the liver injury.
d) **Dose Dependency**: The risk of corticosteroid-induced liver injury is **dose-dependent**, with high doses (e.g., intravenous methylprednisolone) carrying the greatest risk of acute liver injury. These high doses may overwhelm hepatic detoxification pathways and increase susceptibility to mitochondrial dysfunction and oxidative damage.
e) **Withdrawal Effects**: Hepatic complications can also arise upon abrupt withdrawal of corticosteroid therapy, particularly in patients with underlying liver conditions. The cessation of corticosteroids can trigger a rebound inflammatory response, leading to acute exacerbation of preexisting liver damage [43], [44].

**Clinical Manifestations of Corticosteroid-Induced DILI:**

a) **Acute Liver Injury**: High doses of corticosteroids, particularly intravenous administration, are associated with acute hepatocellular injury, presenting with elevated levels of **alanine aminotransferase (ALT)** and **aspartate aminotransferase (AST)**. In severe cases, this can progress to **acute liver failure**.
b) **Hepatomegaly and Steatosis**: Chronic corticosteroid use is linked to the accumulation of glycogen (glycogenosis) or fat (steatosis) within hepatocytes, resulting in an enlarged liver. These effects are typically reversible upon dose reduction or discontinuation of therapy.
c) **Reactivation of Preexisting Conditions**: Corticosteroid use may exacerbate underlying liver conditions, such as viral hepatitis, or trigger autoimmune responses, leading to liver injury. These effects are mediated through immune modulation and should not be confused with the direct mechanisms of DILI [43], [44].

### 4.7 Isoniazid (INH)

Isoniazid (INH) is a first-line treatment for tuberculosis (TB) but is also a well-known cause of drug-induced liver injury (DILI). Acute INH-induced liver injury typically presents as elevated aminotransferase levels (ALT and AST) and, in some cases, jaundice. This form of injury is generally reversible, with liver function normalizing upon discontinuation of therapy. The hepatotoxicity of INH is attributed to its metabolism and the formation of reactive intermediates. INH undergoes bioactivation via hepatic cytochrome P450 enzymes (primarily CYP2E1), generating toxic metabolites, including acetylhydrazine (AcHz) and hydrazine (Hz). These reactive species induce **oxidative stress**, **mitochondrial dysfunction**, and **lipid peroxidation**, leading to hepatocyte damage.

Several factors increase the likelihood of INH-induced hepatotoxicity, including advanced age, alcoholism, malnutrition, Asian racial background, chronic hepatitis B or C, cirrhosis, and genetic variations such as slow acetylation status due to polymorphisms in the N-acetyltransferase 2 (NAT2) enzyme. Slow acetylators accumulate higher levels of AcHz, worsening liver injury. Furthermore, co-administration of rifampicin induces CYP2E1, accelerating INH metabolism and increasing the production of toxic intermediates, thereby exacerbating hepatotoxicity. In most cases, INH causes mild and transient liver injury. However, in a subset of patients, it may progress to severe liver damage or acute liver failure if not promptly identified and managed. Regular monitoring of liver function tests during therapy and discontinuation of INH in cases of significant hepatic injury are essential to prevent severe outcomes [45], [46], [47], [48].

### 4.8 Tetracyclines

Intravenous administration of tetracycline at high doses may cause fatty liver disease resulting in severe liver dysfunction, and acute liver failure and may be fatal. Tetracycline may cause liver damage by inhibiting mitochondrial protein synthesis and hepatocyte lipid metabolism [49], [50].

### 4.9 Halothane

Halothane is a powerful, volatile anesthetic gas that is associated with idiosyncratic liver damage. It causes a slight elevation of serum aminotransferase levels within 1–2 weeks after halothane anesthesia. The incidence of severe liver damage with halothane is about 1 out of 15,000 cases after initial exposure while after repeated exposure incidence is about 1 out of 1000 cases. This injury is characterized by a rapid increase in serum aminotransferase levels and the development of jaundice within 2–14 days after halothane use [51], [52]. Patient may be found with elevated alkaline phosphatase levels, bilirubin and eosinophilia. It is believed that the mechanism of halothane causing liver damage is due to an immune allergic reaction which leads to the formation of reactive halothane intermediates. Trifluoro acetylate hepatic proteins (metabolites from halothane biotransformation) are known to trigger immunogenic and cytotoxic reactions in the liver. About 60–80 % of halothane is excreted unchanged into the lung while the remaining bio is transformed into reactive anesthetic intermediates by the hepatic microsomal enzyme CYP 2E1 [53]. Antibodies against trifluoroacetate proteins are frequently seen in patients with halothane hepatitis. On the other hand, the histologic and clinical pattern of halothane-induced liver injury is similar to that of chloroform-induced liver injury, but with milder centrilobular necrosis, suggesting the possibility of direct injury from hazardous intermediates of halothane reductive metabolism [54].

## 5 Diagnosis of drug-induced liver injury

### 5.1 Biochemical tests

**Fig. 2:**
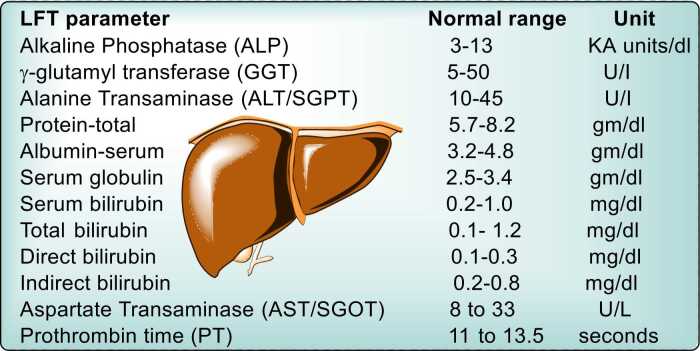
Normal values of liver function parameters [58], [59], [60], [61], [62].

Liver function tests (LFTs) (Fig. 2) are essential for evaluating liver health and diagnosing liver diseases. Key biochemical markers include bilirubin, aspartate transaminase (AST), alanine transaminase (ALT), alkaline phosphatase (ALP), and gamma-glutamyl transpeptidase (GGT). Additional tests, such as prothrombin time, alpha-fetoprotein (AFP), and galactose tolerance, provide valuable information about liver function and specific liver conditions [55], [56], [57].

**Fig. 3:**
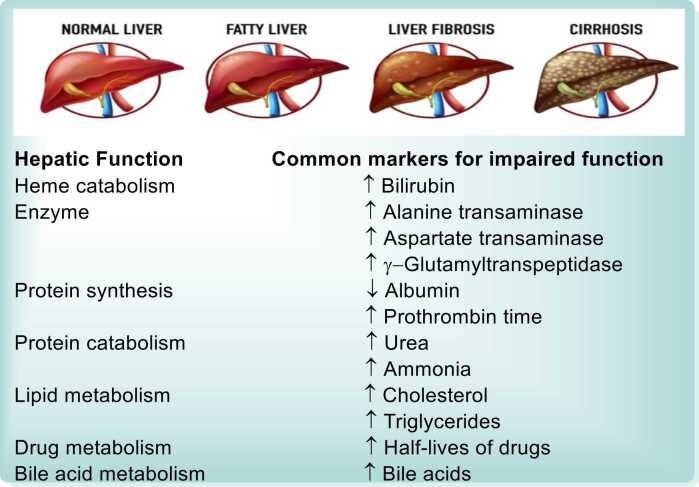
A list of liver functions and the common markers in plasma for impaired function.
• **Alkaline Phosphatase (ALP)**: Elevated ALP levels indicate biliary obstruction (obstructive or post-hepatic jaundice). This elevation is often accompanied by increased serum bilirubin. The normal range for ALP is 3–13 KA units/dl [30].
• **Gamma-Glutamyl Transpeptidase (GGT)**: GGT testing is useful in all forms of liver disease. It is particularly helpful in differentiating the source of ALP elevation, as a concurrent increase in GGT suggests a hepatic origin, whereas isolated ALP elevation may indicate a bone-related cause.
• **Prothrombin Time (PT)**: Prothrombin time measures plasma clotting factor concentrations, which are synthesized by the liver. A prolonged PT indicates a decline in liver function and is a valuable marker in assessing acute and chronic liver damage.
• **Alpha-Fetoprotein (AFP)**: AFP serves as a biomarker for detecting liver tumors (e.g., hepatocellular carcinoma) and germ cell tumors. Elevated AFP levels are often indicative of malignant liver disease.
• **Galactose Tolerance Test**: This test assesses the liver's ability to metabolize galactose. Elevated blood galactose levels are seen in hepatocellular damage, such as infective hepatitis and cirrhosis. This test is useful for evaluating liver metabolic function [63], [64], [65].

• **AST/ALT Ratio**: The AST/ALT ratio typically hovers around 0.8 in healthy individuals. This ratio increases to greater than 2 in conditions like myocardial infarction, cirrhosis, and alcoholic hepatitis. Conversely, the ratio is less than 1 in cases of acute hepatocellular injury and cholestasis, as ALT levels are more elevated than AST. (See Fig. 3)

### 5.2 Serological markers

Serological markers are used to detect hepatitis A, B, and C and other viruses such as the Epstein-Barr virus. The positive findings of these infections can predispose and precipitate the DILI and suggest the physician inspect the probable drug-induced injuries [66].

### 5.3 Radiological investigation

These investigations can be useful for checking/detecting bile obstruction, mass, or strictures. Radiological tests such as computed tomography, endoscopic retrograde cholangiopancreatography (ERCP), percutaneous cholangiograms, and ultrasound are used to detect these possible risk factors.

### 5.4 Liver biopsy

Liver biopsy is helpful for the characterization of lesions like the distribution of micro vesicular fat droplets [63], [64].

### 5.5 Biomarkers of drug-induced liver injury

In DILI, serum markers play a vital role in diagnosing and monitoring liver damage. Commonly measured markers include **total bilirubin (TBIL)**, which reflects bilirubin metabolism and excretion, and enzymes such as **alanine aminotransferase (ALT)** and **aspartate aminotransferase (AST)**, which are indicators of hepatocellular injury. **Alkaline phosphatase (ALP)** and **gamma-glutamyl transferase (GGT)** are used to assess cholestasis and biliary obstruction.

Additionally, mitochondrial macromolecules—**glutamate dehydrogenase (GLDH)** and **mitochondrial DNA (mtDNA)**—are significantly increased during DILI. Both GLDH and mtDNA are located in the mitochondrial matrix and are released when severe mitochondrial damage causes the rupture of mitochondrial membranes [67], [68], [69]. Some additional biomarkers include total bilirubin (TBIL), ALT, AST, ALP, and GGT are known as Traditional markers found during DILI. Similarly, Serum markers include sorbitol dehydrogenase (SDH), keratin 18 (K18), glutathione-S-transferase alpha (GTSα), osteopontin (OPN), anti-CYP450 antibodies and GLDH. Histological markers include*:* Fatty acid binding protein 1, high mobility group box-1 protein, cadherin 5, acetylated-HMGB1, micro RNA examples: mir-122, mir-192, mir-6, mir-320–3p, mir-1–3p and mir-877–5p. The genetic testing includes HLA-B* 57:01 linked to abacavir, DRB1 * 07 and DQA1 * 02 linked to ximelagatran, GSTT1, GSTM1, GSTP1, and HLA-DQB* 05/* 05 linked to antituberculosis medications, etc [70], [71], [72].

## 6 Treatment and management

Managing DILI involves a multifaceted approach, and non-pharmacological strategies play a crucial role. It's crucial to note that the treatment of drug-induced liver injury may require a collaborative effort involving healthcare providers from various disciplines including hepatologists, pharmacists, nutritionists, and mental health professionals. Some of the non-pharmacological measures to manage the drug-induced liver injury are:

### 6.1 Identification and cessation of the culprit medication

Culprit drugs need to be promptly identified and discontinued. Withdrawal of the offending drug is the primary and most effective non-pharmacological intervention. However, the dose adjustment of the drug before the initiation of the therapy is always a wise choice [73], [74].

### 6.2 Supportive care

Provide supportive care to the patients to address symptoms and complications. Supportive measures may include hydration, nutrition support and management of electrolyte imbalances [75].

### 6.3 Liver function monitoring

Daily monitoring of liver function tests to find the progress of liver injury and to guide the clinician in clinical decision-making is the wisest decision while designing the therapy. Close observation of clinical signs and symptoms of hepatic dysfunction is mandatory in suspected cases [76].

### 6.4 Complete abstinence from alcohol and nutritional support

Encourage complete abstinence from alcohol, as it can exacerbate liver damage and hinder recovery. Instead, ensure proper nutrition to support liver function and regeneration. Consider consultation with a nutritionist to optimize the patient's dietary intake [77], [78].

### 6.5 Rest and patient education

Advocate for adequate rest to facilitate recovery and minimize additional stress on the liver. Modify activities as needed to prevent further injury or strain. Educate the patient about the importance of compliance with medical advice and abstinence from potentially hepatotoxic substances, including over-the-counter medications.

### 6.6 Psychosocial support and regular monitoring

Provide psychological support to help the patient cope with the emotional and mental impact of the liver injury. Consider involving a mental health professional if needed. Schedule regular follow-up appointments to monitor liver function and assess overall recovery. Adjust the management plan based on the patient's progress and any changes in liver function tests [79].

### 6.7 Some drug-specific measures to treat the DILI

#### 6.7.1 Acetaminophen

There are minor elevations of aminotransferase that occur during chronic therapy with acetaminophen and rarely have symptoms. It can be resolved either by discontinuation or continuation of the same dose of acetaminophen. An overdose of acetaminophen may cause acute liver damage, and hepatic failure, which may be fatal or require a liver transplant. Further, acute liver damage from acetaminophen can be done by repletion of glutathione levels which can be accomplished with n-acetylcysteine (NAC). It is available in both oral as well as intravenous forms and should be administered immediately upon diagnosis of acetaminophen overdose [80], [81], [82], [83], [84].

#### 6.7.2 NSAID (Non-Steroidal Anti-Inflammatory Drugs)

Ibuprofen: Ibuprofen's liver damage severity ranges from no symptomatic alterations in acute liver failure, acute cholestatic hepatitis and increases in serum aminotransferase levels and there is an immediate need to perform liver transplantation. Ibuprofen use has led to several instances of chronic vanishing bile duct syndrome. Other propionic acid NSAIDs, such as naproxen, oxaprozin, and fenoprofen, should probably be avoided, and patients starting other NSAIDs should be monitored. A current study has demonstrated that compounds like shikonin and celastrol can attenuate some NSAID-induced liver toxicities [84], [85].

#### 6.7.3 Aspirin

Aspirin, when taken in high doses, may cause liver injury, which is typically mild and self-limited. Symptoms resembling liver injury are common but generally non-specific and mild. Supportive therapies, such as **20 % glucose infusions**, may help stabilize liver and brain function during episodes of transient mitochondrial dysfunction, though this is not a specific treatment for aspirin-induced DILI. Alanine aminotransferase (ALT) levels often return to normal within a few days of discontinuing the drug. Ghasemkhani et al. [87] reported the protective effects of **Shilajit** in a mouse model of aspirin-induced hepatotoxicity; however, clinical evidence supporting its efficacy is lacking [86].

#### 6.7.4 Chlorpromazine

There is no need to modify the dose or to discontinue the therapy because the serum aminotransferase levels that occur are usually self-limited. Patients who have experienced liver damage from chlorpromazine may also be sensitive to other phenothiazines, but they typically tolerate atypical antipsychotics. Treatment with ursodiol (12–15 mg/kg/day) may be beneficial for patients with symptomatic cholestasis [87].

#### 6.7.5 Halothane

The severity might be low and temporary without any symptoms or other signs of liver damage, symptomatic and acute like hepatitis, self-limited, or severe like acute hepatic failure. Patients who have hepatitis brought on by halothane should be warned not to use fluorinated hydrocarbon anesthetics such as isoflurane, enflurane, desflurane, or sevoflurane in the future. Guidelines regarding the use of halothane should be followed before the use of this medicine [88].

#### 6.7.6 Isoniazid

There is no definite level at which the administration of isoniazid should be stopped & if liver test monitoring is effective in avoiding hepatotoxicity. The immediate discontinuation of isoniazid should be done if the person has fatigue, nausea, jaundice or poor appetite accompanied by elevations in the liver enzyme.

#### 6.7.7 Methotrexate

Before starting methotrexate therapy, a pretreatment evaluation, including baseline liver function tests and assessment for risk factors such as pre-existing liver disease or alcohol use, is essential to prevent liver damage. The use of ursodeoxycholic acid may help in alleviating the symptoms and normalizing the abnormal parameters [89].

#### 6.7.8 Rifampin

Rifampicin-induced DILI may be in symptomatic and non-symptomatic, self-limited hepatitis to severe liver failure and rarely death. The termination of rifampin usage is advised for the complete recovery. The recovery is usually rapid from this condition [90].

#### 6.7.9 Tetracycline

It can result in acute fatty liver which has a high fatality rate and is managed with intensive care & attention in case of lactic acidosis, an infusion of glucose IV 20 % & bicarbonate should be provided. Idiosyncratic acute liver injury is very rare & resolves after the drug is removed [90].

## 7 Indian scenario for drug-induced liver disease (DILI)

Drug-induced liver injury (DILI) is a major cause of acute liver failure worldwide and is responsible for a significant proportion of medication withdrawals post-marketing. While the incidence and etiology of DILI differ regionally, traditional medicines play a prominent role in countries like India and China, whereas nutritional supplements and herbal remedies are more frequently implicated in the West [12].

### 7.1 Global context of DILI

In the US and Europe, approximately 15 % of acute liver failure cases are attributed to DILI, often caused by commonly prescribed medications and supplements. Studies in Africa highlight anti-tuberculosis (anti-TB) and antiretroviral drugs as primary contributors due to the high prevalence of TB and HIV in the region. This regional variation reflects the drugs most widely used in specific populations [88].

### 7.2 DILI in India

In India, DILI is primarily linked to the widespread use of anti-TB medications, complementary therapies, and traditional medicines. According to a prospective study conducted between 2013 and 2018 involving 1288 patients:

• **46.4 %** of cases were associated with anti-TB drugs.
• **13.9 %** involved complementary therapies.
• **8.1 %** were linked to anti-epileptic medications.
• Other causes included statins, NSAIDs, antiretroviral therapies, and non-TB antibiotics.

Nearly 32.4 % of these cases were severe, with 68 % presenting with jaundice and requiring hospitalization. Although Ayurvedic and herbal medicines (AHMs) are often implicated in DILI, only a small percentage of these therapies cause significant liver damage. However, the lack of regulation and labeling of multi-ingredient preparations in traditional medicine poses challenges for identifying hepatotoxic agents. Contamination with heavy metals or chemical substances further complicates safety assessments [91], [92].

### 7.3 Challenges with traditional medicines

Traditional Indian medicine systems like Ayurveda, Unani, Siddha, and homeopathy are extensively used, often in combination with allopathic therapies. The lack of proper monitoring and regulation of these therapies increases the risk of DILI. Many of these preparations contain unlabeled ingredients or contaminants, such as heavy metals, which can exacerbate hepatotoxicity. Enhanced monitoring, regulation, and early liver biopsy in at-risk patients can mitigate the impact of these therapies on liver health [93], [94], [95].

## 8 Prevention

Due to the limited treatment options for drug-induced liver injury (DILI), the emphasis has shifted towards prevention. During the drug discovery process, a candidate drug needs to meet stringent screening criteria before advancing to clinical trials. Evaluating the individual risk factors of patients before starting a known hepatotoxic medication can help prevent genetically associated adverse reactions. As personalized medicine becomes more prevalent, incorporating these preventive measures could become standard practice. As the healthcare industry advances towards personalized medicine, incorporating risk assessment and prevention strategies for drug-induced liver injury (DILI) is becoming increasingly important. Restricting the availability of known hepatotoxic drugs is another effective measure to prevent misuse. For example, the United Kingdom introduced legislation in 1998 to limit the pack sizes and sales of paracetamol to 16 tablets in regular stores and 32 in pharmacies. This policy led to a 43 % reduction in paracetamol-related deaths and a 61 % decrease in liver transplants due to paracetamol overdose from 1998 to 2009 [96], [97], [98], [99].

Continuous monitoring of patients who are on long-term or combination drug therapies can also help prevent DILI-related acute liver failure (DILI-ALF) by detecting asymptomatic cases early before permanent damage occurs. Additionally, point-of-care testing for alanine aminotransferase (ALT), similar to finger-prick glucose monitoring, has been proposed as a practical system for monitoring patients at risk of hepatotoxicity [100], [101]. This approach could facilitate timely interventions and improve patient outcomes measures could become standard practice.

Elsewhere, clinical monitoring is recommended for patients taking isoniazid. Physicians can prevent the development of drug-induced liver injury (DILI) by avoiding or substituting known hepatotoxic agents. This practice is particularly important for patients with chronic liver disease, where the use of drugs such as valproic acid, methotrexate, ketoconazole, and tolvaptan is discouraged [102]. Additionally, reporting DILI incidents is crucial for identifying patterns, enhancing understanding, and providing a comprehensive overview of DILI. This information is vital for developing preventive strategies and improving patient safety.

## 9 Clinical pharmacist’s interventions in DILD

Clinical pharmacists play a crucial role in the management of DILD by optimizing pharmacotherapy, educating patients and collaborating with healthcare teams to ensure the best outcomes. DILD can arise from a variety of mechanisms, including direct hepatotoxicity, idiosyncratic reactions and immune-mediated responses. Direct hepatotoxicity occurs when a drug or its metabolites cause direct damage to liver cells. Idiosyncratic reactions are unpredictable and not dose-dependent, often linked to genetic and environmental factors. Immune-mediated liver injury involves an immune response triggered by the drug, leading to liver inflammation and damage [103]. Factors such as genetic predisposition, underlying liver conditions, polypharmacy, and patient-specific characteristics (e.g., age, sex, comorbidities) contribute to the risk and severity of DILD. Clinical pharmacists are essential in preventing, identifying, and managing DILD through various interventions. These include medication review and reconciliation, risk assessment, patient education, implementation of monitoring protocols, pharmacogenomics application, and collaborative care with other healthcare professionals [104].

### 9.1 Medication review and reconciliation

One of the primary interventions by clinical pharmacists is conducting thorough medication reviews and reconciliation. This process involves evaluating the patient’s complete medication list to identify potential hepatotoxic agents and assessing their necessity and safety. Clinical pharmacists can recommend discontinuation, substitution, or dosage adjustment of hepatotoxic drugs to mitigate the risk of DILD.

### 9.2 Risk assessment and stratification

Risk assessment involves identifying patients at high risk of developing DILD and implementing strategies to mitigate this risk. Clinical pharmacists can use tools such as the Roussel Uclaf Causality Assessment Method (RUCAM) to evaluate the likelihood of drug-induced liver injury. By stratifying patients based on their risk, pharmacists can tailor interventions accordingly [105], [106].

### 9.3 Patient education

Educating patients about the risks of DILD and the importance of medication adherence and monitoring is a crucial role of clinical pharmacists. Patients need to be informed about the signs and symptoms of liver injury, the importance of routine liver function tests (LFTs), and the potential interactions of prescribed medications with over-the-counter drugs and dietary supplements.

### 9.4 Implementation of monitoring protocols

Regular monitoring of liver function tests is essential for patients receiving potentially hepatotoxic medications. Clinical pharmacists can establish and implement monitoring protocols, ensuring that appropriate tests are conducted at baseline and periodically during treatment. Pharmacists also interpret LFT results and recommend necessary adjustments to therapy based on these findings [107].

### 9.5 Pharmacogenomics

Pharmacogenomics involves studying how genetic variations affect an individual’s response to drugs. Clinical pharmacists can utilize pharmacogenomic data to identify patients at higher risk of DILD and personalize medication regimens to enhance safety and efficacy. Several genetic polymorphisms are associated with increased susceptibility to DILD, such as variants in the HLA and CYP450 genes. The integration of pharmacogenomic testing into clinical practice allows pharmacists to make informed decisions about drug selection and dosing, reducing the risk of liver injury [108], [109].

### 9.6 Collaborative care

Collaborative care involves clinical pharmacists working alongside physicians, nurses, and other healthcare professionals to manage DILD. This multidisciplinary approach ensures comprehensive care, encompassing all aspects of patient management, from medication selection to monitoring and follow-up.

### 9.7 Impact on healthcare costs

Clinical pharmacist interventions not only improve patient outcomes but also reduce healthcare costs associated with DILD. By preventing severe liver injury and reducing hospitalizations, pharmacist-led interventions can lead to substantial cost savings [110].

## 10 Evidences and implications

Drug-induced hepatotoxicity is a leading cause of drug withdrawal from global markets. Acute liver failure (ALF) is a notable example of drug-induced hepatotoxicity and occurs with alarming frequency. DILI is a significant factor in both drug recalls and cases of ALF. Despite the distinctions between intrinsic and idiosyncratic DILI, hepatotoxicity remains a global concern. Further examination of the dose-response relationship might reveal similarities between these two types of reactions. In cases of idiosyncratic hepatotoxicity, the liver might not typically be a target for toxicity because the standard dose-response curve for hepatotoxicity is below the lethal dose for most users. However, during an inflammatory episode in the liver, the hepatotoxic dose might fall within the therapeutic range, leading to a spontaneous toxic response. This suggests that, under certain conditions, idiosyncratic hepatotoxicity can manifest like intrinsic hepatotoxicity, highlighting the complexity of predicting and managing drug-induced liver injuries.

As previously indicated, a growing body of literature has demonstrated that xenobiotic-induced adverse effects in the liver are exacerbated in a diseased organ. Consequently, patients with metabolically compromised livers face an increased health risk when exposed to toxic compounds, including drugs. If validated, this observation has significant implications for preclinical hepatotoxicity testing strategies in drug development and the assessment of drug-induced liver injury (DILI). It underscores the need to evaluate drug safety not only in models representing healthy livers but also in those with altered metabolism. For instance, clinically asymptomatic metabolic-associated fatty liver disease (MASLD) might render patients on medication at a higher risk for developing liver injury, yet this cohort is often underrepresented in clinical safety studies. Jinzhang et al., 2020 [111] reported that out of 305 investigated trials, one-third were at high risk of bias, primarily because the clinical populations used were not appropriate for the trials examined. It is reasonable to assume that similar concerns apply to heavy drinkers, who may be more susceptible to developing acute DILI.

The implication is that current preclinical and clinical testing strategies might not adequately account for the variability in liver health among different populations. This gap could lead to an underestimation of the hepatotoxic potential of new drugs in patients with underlying liver conditions. Therefore, it is crucial to include diverse liver health models in safety assessments to better predict and mitigate the risks of DILI in vulnerable populations.

Evidence suggests that a significant consideration emerging from the literature is the shortage of animal models that accurately capture idiosyncratic drug-induced liver injury (iDILI). However, several promising models, such as the Inflammagen model [97], [98] and the Uetrecht-Pohl model [105] show potential. The prevailing view of iDILI is that drugs require transformation into metabolites by phase I enzymes, followed by phase II conjugation. This biotransformation produces chemically reactive or unstable metabolites capable of causing cellular dysfunction and liver injury. This understanding underscores the importance of recognizing significant intra-species differences in metabolism. Although common experimental animal models, particularly mice, express homologs of the major human cytochrome P450 (CYP450) enzymes, these homologs do not always have the same substrate specificity [112]. As a result, the predictivity of human responses based on these models is generally poor. Therefore, fully considering species differences in drug metabolism is critical to improving the accuracy of preclinical safety assessments and better predicting potential hepatotoxicity in humans.

The lack of specific biomarkers makes distinguishing drug-induced liver injury (DILI) from other hepatic conditions challenging. This issue is compounded by the fact that DILI diagnosis primarily relies on excluding other potential causes. Collaborative efforts to identify new biomarkers are ongoing, but they are still in the clinical exploration phase. Studies focusing on the incidence of DILI over time indicate a higher occurrence of DILI with the increased use of chemotherapy drugs. Although international consortium thresholds have provided some assistance, more precise assessment and reporting methods are necessary. Most DILI cases are self-limiting and resolve upon withdrawal of the offending drug, but treatments for more severe reactions remain limited. Large studies have reported DILI mortality rates of approximately 5–10 %, with liver transplantation often considered when symptoms progress to acute liver failure (ALF) [113]. There is no standardized clinical guidance for DILI treatment and most treatments are nonspecific. Current causality assessment models can be ambiguous, often lacking comprehensive data to support some of their components.

Research indicates that while female gender and increased age are not overall risk factors for DILI, these demographics are more likely to experience certain types of injury and have a higher risk of mortality. The identification and implementation of specific biomarkers, along with improved assessment and reporting methods, are essential for advancing the understanding and management of DILI. Drug characteristics such as lipophilicity and molecular structure have been identified as risk factors for the development of drug-induced liver injury (DILI). These findings have led to the enforcement of strict hepatotoxicity criteria throughout the drug discovery process. As personalized medicine becomes more prevalent, genetic screening prior to the administration of potentially hepatotoxic drugs may become necessary [114]. Additionally, point-of-care testing for liver enzyme activity elevations could facilitate early diagnosis and monitoring.

Despite advancements, much remains unknown about DILI. Future research is essential to aid physicians in managing this challenging diagnosis, which often relies on exclusion of other conditions. Due to the lack of specific diagnostic biomarkers, DILI assessment largely depends on subjective expert consensus. The Roussel Uclaf Causality Assessment Method (RUCAM) scale is a valuable tool for causality assessment, offering a standardized approach to evaluate DILI. Over the past decade, significant advances in nanomedicine have shown promise in preventing and treating liver damage, including DILI. Targeted therapeutic delivery directly to the site of injury is an attractive strategy. Moreover, the growing body of research on the interactions between gut microbes and host cells may open new avenues for developing therapeutic applications to prevent and treat acute DILI.

## 11 Conclusion

The literature and existing causality assessment tools highlight the need for well-designed mechanistic studies. Numerous investigations have noted a link between DILI and polymorphisms in certain HLA genotypes and CYP450 enzymes. However, more research is urgently needed to better understand the mechanisms underlying DILI and idiosyncratic DILI (iDILI). Advancements in understanding the molecular and genetic factors contributing to DILI will enhance the ability to predict and prevent this adverse drug reaction. Collaborative efforts to develop specific biomarkers, improve animal models and refine causality assessment tools are crucial. Such efforts will ultimately lead to more accurate diagnosis, better prevention strategies and improved treatment options for patients at risk of DILI.

## Source of funding

None.

## CRediT authorship contribution statement

**Tiwari Vatsalya:** Writing – original draft, Formal analysis, Data curation, Conceptualization. **Chand Sharad:** Writing – review & editing, Writing – original draft, Supervision, Formal analysis, Data curation, Conceptualization. **Singh Rohit:** Writing – review & editing, Formal analysis, Data curation, Conceptualization. **Sah Sujit Kumar:** Writing – original draft, Validation, Formal analysis. **Mishra Vaibhav:** Writing – original draft, Formal analysis, Data curation, Conceptualization. **Dikkatwar Manoj:** Writing – original draft, Supervision, Methodology, Formal analysis. **Shandily Shrishti:** Writing – original draft, Formal analysis, Data curation, Conceptualization. **Albert Jessielina:** Writing – original draft, Formal analysis, Data curation.

## Declaration of Generative AI and AI-assisted technologies in the writing process

During the preparation of this work the author(s) used ChatGPT in order to improve the punctuation. After using this tool/service, the author(s) reviewed and edited the content as needed and take(s) full responsibility for the content of the publication.

## Declaration of Competing Interest

The authors declare that they have no conflicts of interest.

## Data Availability

No data was used for the research described in the article.
