# Corrigendum to “Insights into medication-induced liver injury: Understanding and management strategies” [Toxicol. Rep. 14 (2025) 101976]

**DOI:** 10.1016/j.toxrep.2025.102068

**Published:** 2025-06-16

**Authors:** Vatsalya Tiwari, Shrishti Shandily, Jessielina Albert, Vaibhav Mishra, Manoj Dikkatwar, Rohit Singh, Sujit Kumar Sah, Sharad Chand

**Affiliations:** a Amity Institute of Pharmacy, Amity University, Noida, Uttar Pradesh, India; b DY Patil University School of Pharmacy, DY Patil (Deemed to be University), Nerul, Navi Mumbai, Maharashtra 400706, India; c Department of Pharmaceutical Sciences, School of Health Sciences and Technology, Dr. Vishwanath Karad MIT World Peace University, Pune, Maharashtra 411038, India

The authors regret for the error occurred in the highlights statement in the published article *“The spectrum of DILI encompasses around 1/5th of total population prescribed with medications.”.*

This needs to be corrected as *“The spectrum of DILI encompasses around 14–19 cases per 100,000 population.”*

The authors would like to apologise for any inconvenience caused.

